# Targeted Regulation of *AhGRF3b* by ahy-miR396 Modulates Leaf Growth and Cold Tolerance in Peanut

**DOI:** 10.3390/plants14203203

**Published:** 2025-10-18

**Authors:** Xin Zhang, Qimei Liu, Xinyu Liu, Haoyu Lin, Xiaoyu Zhang, Rui Zhang, Zhenbo Chen, Xiaoji Zhang, Yuexia Tian, Yunyun Xue, Huiqi Zhang, Na Li, Pingping Nie, Dongmei Bai

**Affiliations:** 1 Institute of Industrial Crops, Shanxi Agricultural University, Taiyuan 030031, China; sxndjzszx@sxau.edu.cn (X.Z.); sxndjzstyx@sxau.edu.cn (Y.T.); sxndjzsxyy@sxau.edu.cn (Y.X.); sxndjzszhq@sxau.edu.cn (H.Z.); sxndjzsln@sxau.edu.cn (N.L.); 2 College of Plant Protection, Shanxi Agricultural University, Taigu 030801, China; z20223416@stu.sxau.edu.cn (Q.L.); 20233423@stu.sxau.edu.cn (X.Z.); 3 College of Life Sciences, Zaozhuang University, Zaozhuang 277160, China; lixiaohong@uzz.edu.cn (X.L.); yuanjuanjuan@uzz.edu.cn (H.L.); 4 College of Agronomy, Shanxi Agricultural University, Taigu 030801, China; 202430284@stu.sxau.edu.cn (R.Z.); 202430118@stu.sxau.edu.cn (Z.C.); s20222136@stu.sxau.edu.cn (X.Z.)

**Keywords:** peanut, growth-regulating factors (GRFs), miR396, leaf development, cold tolerance

## Abstract

Peanut (*Arachis hypogaea* L.) is an important oil and cash crop, but its growth and productivity are severely constrained by low-temperature stress. Growth-regulating factors (GRFs) are plant-specific transcription factors involved in development and stress responses, yet their roles in peanut remain poorly understood. In this study, we identified *AhGRF3b* as a direct target of ahy-miR396 using degradome sequencing, which demonstrated precise miRNA-mediated cleavage sites within the *AhGRF3b* transcript. Expression profiling confirmed that ahy-miR396 suppresses *AhGRF3b* via post-transcriptional cleavage rather than translational repression. Functional analyses showed that overexpression of *AhGRF3b* in *Arabidopsis thaliana* promoted leaf expansion by enhancing cell proliferation. Specifically, leaf length, width, and petiole length increased by 104%, 22%, and 28%, respectively (*p* < 0.05). Under cold stress (0 °C for 7 days), transgenic lines (OE-2 and OE-6) exhibited significantly better growth than Col-0, with fresh weight increased by 158% and 146%, respectively (*p* < 0.05). Effect size analysis further confirmed these differences (Cohen’s d = 11.6 for OE-2 vs. Col-0; d = 6.3 for OE-6 vs. Col-0). Protein–protein interaction assays, performed using the yeast two-hybrid (Y2H) system and 3D protein–protein docking models, further supported that AhGRF3b interacts with Catalase 1 (AhCAT1), vacuolar cation/proton exchanger 3 (AhCAX3), probable polyamine oxidase 4 (AhPAO4), and ACT domain-containing protein 11 (AhACR11), which are involved in reactive oxygen species (ROS) scavenging and ion homeostasis. These interactions were associated with enhanced CAT and PAO enzymatic activities, reduced ROS accumulation, and upregulation of stress-related genes under cold stress. These findings suggest that the ahy-miR396/*AhGRF3b* module plays a potential regulatory role in leaf morphogenesis and cold tolerance, providing valuable genetic resources for breeding cold-tolerant peanut varieties.

## 1. Introduction

Peanut (*Arachis hypogaea* L.) is one of the most important oilseeds and economic crops globally, providing both plant oils and protein resources for human consumption while also playing a crucial role in sustainable agriculture [1]. However, during peanut growth and development, low-temperature stress severely restricts yield and quality. Low temperatures inhibit seed germination and seedling growth, disrupt photosynthesis, and cause metabolic disorders, which in severe cases may even lead to complete crop failure, posing a significant threat to agricultural production and farmers’ income [2]. Therefore, understanding the molecular mechanisms underlying cold tolerance in peanuts and identifying key genes are crucial for developing cold-tolerant varieties, ensuring stable yields and quality.

In plants, cold tolerance is largely regulated through canonical pathways, most notably the C-repeat binding factor/dehydration-responsive element-binding (CBF/DREB) transcriptional cascade, which activates downstream cold-responsive (COR) and late embryogenesis abundant (LEA) genes. These genes contribute to membrane stabilization, osmoprotection, and reactive oxygen species (ROS) detoxification, thereby forming the core molecular framework of cold acclimation [2]. With the rapid development of molecular biology and genomics, the central role of non-coding small RNAs (sRNAs) in plant stress responses has become increasingly recognized. sRNAs are a class of non-coding molecules 21–24 nucleotides in length, mainly including microRNAs (miRNAs) and small interfering RNAs (siRNAs). These molecules regulate gene expression at the post-transcriptional level and play pivotal roles in plant development and responses to environmental stresses. Environmental factors such as temperature, drought, and salinity can significantly affect the stability and processing of sRNAs [3]. Among them, miR396 has attracted particular attention due to its high conservation and functional diversity in plants [4]. Studies have shown that miR396 regulates leaf development and abiotic stress responses by targeting Growth-regulating factors (GRFs). For example, over-expression of ath-miR396 in *Arabidopsis thaliana* leads to a significant reduction in leaf area and cell number due to the repression of *GRF* activity and a decrease in the expression of cell cycle genes, while over-expression of *GRF* genes can partially rescue the reduced leaf growth phenotype induced by ath-miR396 overexpression [5]. In tobacco, over-expression of Sp-miR396a-5p markedly enhanced tolerance to salt, drought, and low temperature [6], further demonstrating that the miR396–*GRF* regulatory module plays a broad and crucial role in plant adaptation to adverse environments. miR396 regulates *GRF* expression at the molecular level by binding to the *GRF* mRNAs, leading to their cleavage and suppression, which directly impacts the growth and stress responses of the plant.

The *GRF* gene family, a plant-specific transcription factor family, was first identified in rice and named *OsGRF1* [7]. GRF proteins typically contain two conserved domains: the QLQ (Gln-Leu-Gln) domain and the WRC (Trp-Arg-Cys) domain located at the N-terminus. The QLQ domain interacts with the SNH domain of GRF-interacting factors (GIFs) to form a transcriptional co-activator complex, thereby activating the transcription of downstream genes. The WRC domain contains a C3H-type zinc finger motif and a nuclear localization signal, enabling it to bind to cis-acting elements and regulate the spatiotemporal expression of target genes [8,9,10]. These molecular features allow *GRF* genes to play vital roles in plant growth, development, and environmental adaptation, including the regulation of leaf morphogenesis, root apical meristem activity, floral organ differentiation, seed development, hormone signaling, and secondary metabolism [8,11,12,13]

A growing body of research has demonstrated that *GRF* genes exhibit differential expression patterns across species and tissues. In poplar and tea, most *GRF* genes are highly expressed in young leaves [14,15]; in strawberry, *FvGRF3/4/7* and in chickpea, *CaGRF1/4/5/6/7* show higher expression levels in roots [16,17]; in *Astragalus membranaceus*, *AmGRF1/2/3/4/9*, and in strawberry, *FvGRF1* are predominantly expressed in stems [16,18]. In flowers and reproductive structures, *StGRF12/13* in potato [19], *PaGRF5* in apricot [20], and *GhGRF14* in cotton [21] exhibit strong expression. Moreover, in seeds and embryos, *CaGRF2* in chickpea, *CcGRF1/3/6/10* in pigeon pea [17], *PgGRF12/14/16* in ginseng [13], and *SbGRF3/6/7* in sorghum [22] all display significant expression. Collectively, these studies indicate that *GRF* genes play essential regulatory roles in diverse tissues and developmental stages of plants, and have undergone extensive functional diversification during evolution. Importantly, many *GRF* genes, particularly those expressed in young leaves, roots, and stems, are involved in the regulation of growth processes that help plants adapt to environmental stressors, including cold stress.

In terms of abiotic stress responses, the expression of *GRF* genes also exhibits diversity and adaptability. For instance, *PgGRF4, PgGRF6,* and *PgGRF11* in ginseng are significantly upregulated under cold stress [13]; *FvGRF3, FvGRF5,* and *FvGRF7* in strawberry are induced by low temperature [16]; *TaGRF21* and *TaGIF5* in wheat are upregulated under cold conditions [23]; several members including *SlGRF1, SlGRF2*, and *SlGRF3* in tomato show enhanced expression under cold stress [24]; *CsGRF4* and *CsGRF7* in citrus display elevated expression following low-temperature induction [25]; and *PavGRF2, PavGRF3, PavGRF7*, and *PavGRF8* in sweet cherry are activated by cold treatment [26]. These findings suggest that upregulated *GRF* genes are likely involved in promoting cold tolerance through regulating cell proliferation, enhancing stress-responsive gene expression, and modulating metabolic pathways. Moreover, *GRF* genes also respond strongly to salt and drought stresses. For example, *PgGRF7* and *PgGRF16* in ginseng exhibit increased expression under salt stress [13]; *SbGRF1/2/3/6/7* in sorghum are upregulated under drought treatment [22]; *FvGRF6* and *FvGRF8* in strawberry show enhanced expression during drought stress [16]; and *JcGRF1/2/5* in physic nut are upregulated under both drought and salt stresses [27]. Collectively, these findings indicate that *GRF* genes not only play fundamental roles in plant development but also participate broadly in the molecular regulation of plant responses to adverse environmental conditions, including cold stress.

Although *GRF* transcription factors have been systematically studied in various species, their functional analysis in peanut remains relatively limited. Previous studies have found that under low-temperature stress, the expression level of ahy-miR396 in peanut is elevated, while the expression of its target gene *AhGRF3b* is significantly suppressed, indicating that ahy-miR396 negatively regulates peanut cold tolerance by targeting *AhGRF3b* [28]. This suggests that *AhGRF3b* may be an important regulatory factor in peanut’s response to low-temperature stress.

In light of these findings, we further investigated the specific role of *AhGRF3b* in leaf growth and cold tolerance in peanuts. Through gene expression analysis and functional validation, our results show that overexpression of *AhGRF3b* significantly promoted leaf growth and enhanced the plant’s tolerance to low-temperature stress. We further observed that plants overexpressing *AhGRF3b* exhibited better growth under cold conditions compared to the control group, and that the enhanced cold tolerance was associated with the regulation of ROS scavenging pathways and related signaling networks.

## 2. Results

### 2.1. The AhGRF3b Gene Targeted by ahy-miR396

**Figure 1:**
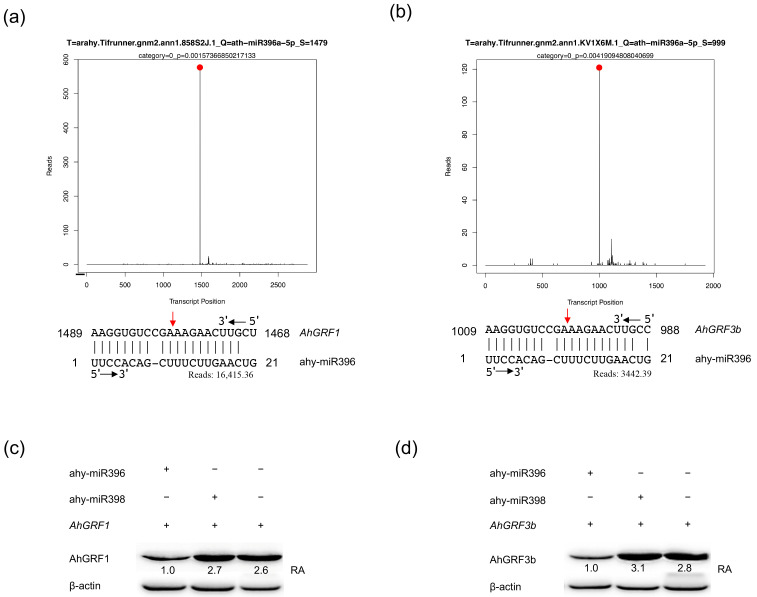
Identification and validation of ahy-miR396 target genes. (**a**,**b**) Degradome sequencing supported that ahy-miR396 cleaves *AhGRF1* (**a**) and *AhGRF3b* (**b**) transcripts at the predicted target sites. The red arrows indicate the cleavage peaks corresponding to the miRNA binding regions at TSlice 1479 for *AhGRF1* (**a**) and TSlice 999 for *AhGRF3b* (**b**), while the red dots mark the specific cleavage sites identified by sequencing. (**c**,**d**) Western blot analysis showing that ahy-miR396 suppresses the protein accumulation of AhGRF1 (**c**) and AhGRF3b (**d**), whereas ahy-miR398 has no significant effect. β-actin served as a loading control. RA: relative abundance.

To elucidate the biological functions of ahy-miR396, high-throughput degradome sequencing was performed. The analysis supported distinct cleavage sites within the mRNA sequences of both *AhGRF1* (Figure 1a) and *AhGRF3b* (Figure 1b), indicating that these genes are direct targets of ahy-miR396. The cleavage patterns exhibited characteristic peaks at the predicted miRNA binding sites, consistent with typical miRNA-mediated degradation. For *AhGRF1*, degradome sequencing identified a predominant cleavage site at nucleotide position 1704 of the CDS, corresponding to the canonical 10th nucleotide relative to the ahy-miR396 5′ end. The miR396 seed region (positions 2–8) showed perfect pairing with the target sequence. This cleavage event was strongly supported by 16,415.36 reads, ranked as the global maximum (Category 0) with highly significant confidence (*p* = 0.00). Similarly, for *AhGRF3b*, a major cleavage site was detected at nucleotide position 1215 of the CDS, also located at the canonical 10th nucleotide position relative to ahy-miR396, with perfect pairing in the seed region. This event was supported by 3442.39 reads, likewise classified as Category 0 (*p* = 0.00). Western blot assays further validated these findings, demonstrating that expression of ahy-miR396 markedly reduced the protein abundance of AhGRF1 (Figure 1c) and AhGRF3b (Figure 1d), confirming post-transcriptional repression through mRNA cleavage. β-Actin was used as the loading control, and band intensities were quantified using ImageJ software (version 1.53t, National Institutes of Health, Bethesda, MD, USA) relative to β-Actin for normalization. The quantified data shown below each blot indicate that the relative protein levels of AhGRF1 and AhGRF3b in the presence of ahy-miR396 were substantially lower than those in the negative control (2.7-fold and 3.1-fold reduction, respectively) and ahy-miR398 groups (2.6-fold and 2.8-fold reduction, respectively), demonstrating the specific repressive effect of ahy-miR396 on these targets. The analysis focused on *AhGRF1* and *AhGRF3b*, while other potential miRNA targets were detected but excluded from our study due to insufficient confidence.

Notably, under low-temperature stress, *AhGRF3b* was significantly upregulated in the cold-tolerant variety WQL30 (9-fold increase at 6 °C for 12 h), whereas *AhGRF1* showed a 4.5-fold increase, suggesting a stronger role of *AhGRF3b* in cold adaptation [28]. Given its pronounced responsiveness to abiotic stress and regulatory significance, *AhGRF3b* was selected as the focal gene for subsequent functional investigations.

### 2.2. Subcellular Localization of AhGRF3b

**Figure 2:**
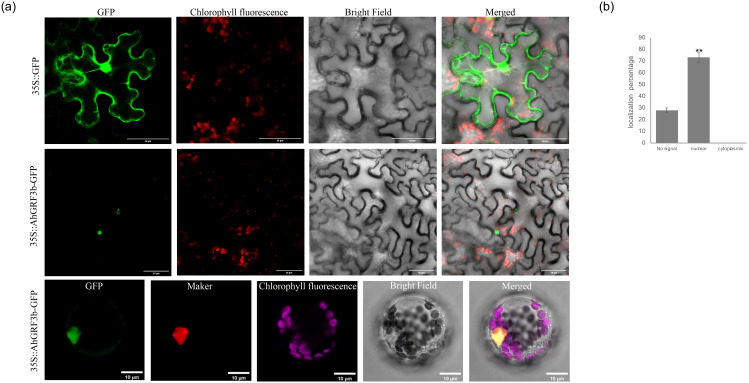
Subcellular localization of the AhGRF3b–GFP fusion protein. (**a**) The subcellular localization of the AhGRF3b–GFP fusion protein was observed in *Arabidopsis* protoplasts and *N. benthamiana* leaves transiently transformed via *Agrobacterium tumefaciens*. Imaging was performed using a confocal laser scanning microscope with a magnification of 63×. Laser settings included an excitation wavelength of 488 nm for GFP fluorescence, with emission collected between 510–530 nm. The images were taken from five independent replicates, each consisting of five fields of view. The fields include bright field (Bright), green fluorescent protein (GFP), chloroplast autofluorescence (Chloroplast), overlap field merged by Bright, GFP, and Chloroplast (Merged), and nucleus marker (NLS-mCherry). Green: Indicates the localization of the AhGRF3b–GFP fusion protein and free GFP protein (green fluorescence). Red (Tobacco cells): Represents chloroplast autofluorescence in tobacco cells (red fluorescence). Red (*Arabidopsis* cells, mCherry): In *Arabidopsis* cells, red corresponds to the nuclear localization marker labeled with red fluorescent protein (mCherry). Gray: Bright field image showing the overall cell structure. Yellow (Merged image): Merged image showing GFP (green) and the nuclear localization marker (red, mCherry), indicating colocalization of the AhGRF3b–GFP fusion protein with the nuclear marker. Purple (*Arabidopsis* cells): Represents chloroplast autofluorescence in *Arabidopsis* cells (red fluorescence). The construct was driven by the 35S promoter to express the AhGRF3b-GFP fusion protein in *Arabidopsis*, using the pCAMBIA1300 vector backbone. The construct was introduced into plants using the floral-dip method. To validate the specificity of the observed localization, control constructs were included: (1) a free GFP construct to assess GFP expression alone, and (2) a nuclear marker (NLS-mCherry) to confirm nuclear localization. Bars represent 10 μm. (**b**) Quantification of AhGRF3b localization: Graph showing the percentage of nuclear, cytoplasmic, and no signal localization of AhGRF3b–GFP in protoplasts and leaves (*n* = 3 biologically independent plants; data are mean ± SD; one-way ANOVA followed by Tukey’s HSD; ** *p* < 0.01).

To establish the subcellular localization of AhGRF3b, the constructed vectors were transiently expressed in *Arabidopsis* protoplasts and in *Nicotiana benthamiana* leaves. The subcellular localization results showed that the GFP signal from the recombinant vector 35S::*AhGRF3b*: GFP was exclusively detected in the nucleus (Figure 2), indicating that AhGRF3b is a nuclear-localized protein. Localization of AhGRF3b-GFP was quantified by calculating the percentage of cells exhibiting nuclear localization versus cytoplasmic signal. The analysis demonstrated that 73% of cells displayed nuclear localization, while 0% exhibited cytoplasmic signal, and about 27% showed no detectable signal. Statistical analysis using one-way ANOVA followed by Tukey’s test confirmed that the difference between nuclear and cytoplasmic localization was statistically significant (*p* < 0.01). AhGRF3b was found to be localized in the nucleus, with negligible cytoplasmic signal, indicating that AhGRF3b functions within the nucleus. These results align with previous reports showing that GRFs are largely nuclear-localized, with little to no evidence of shuttling between the nucleus and cytoplasm.

### 2.3. Physiological Parameter Measurement and Cold Tolerance Analysis of AhGRF3b-Overexpressing Plants

**Figure 3:**
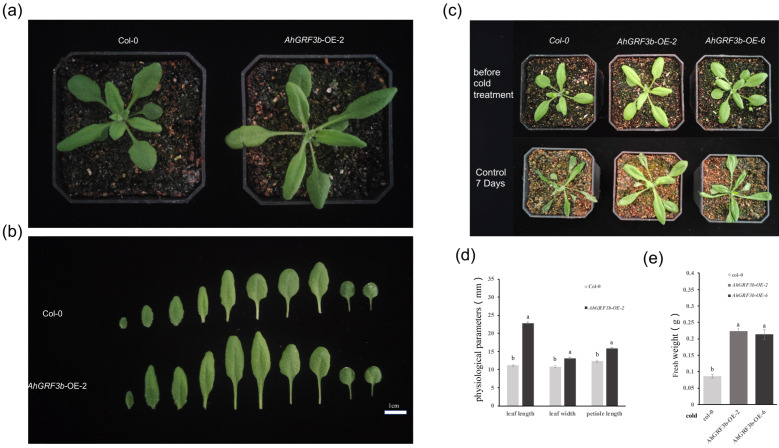
Effect of *AhGRF3b* on leaf morphology and cold tolerance in *Arabidopsis*: (**a**) Comparison of overall plant morphology between wild-type *Arabidopsis* (Col-0) and the transgenic overexpression line *AhGRF3b*-OE-2; (**b**) Comparison of individual leaves from Col-0 and *AhGRF3b*-OE-2, showing differences in leaf size and morphology; (**c**) Phenotypic comparison of Col-0 and two independent transgenic lines (OE-2 and OE-6) before and after 7 days of low-temperature treatment; (**d**) Statistical analysis of leaf physiological parameters (leaf length, leaf width, and petiole length) between Col-0 and *AhGRF3b*-OE-2. Different letters indicate significant differences (*n* = 3 biologically independent plants; data are mean ± SD; one-way ANOVA followed by Tukey’s HSD; *p* < 0.05); (**e**) Fresh weight comparison of Col-0 and the two transgenic lines (OE-2 and OE-6) under low-temperature treatment. Different letters indicate significant differences (*n* = 3 biologically independent plants; data are mean ± SD; one-way ANOVA followed by Tukey’s HSD; *p* < 0.05).

To investigate the effect of *AhGRF3b* on leaf development in *A. thaliana*, phenotypic comparisons were conducted between overexpression lines (*AhGRF3b*-OE) and the wild type (Col-0). The results showed that *AhGRF3b*-OE-2 plants exhibited a larger overall morphology compared with Col-0 (Figure 3a). At the individual leaf level, the leaves of *AhGRF3b*-OE-2 were larger, characterized by increased length, width, and significantly elongated petioles (Figure 3b). Further physiological measurements confirmed that leaf length, leaf width, and petiole length in *AhGRF3b*-OE-2 were all significantly higher than those of Col-0 (*p* < 0.05) (Figure 3d). Specifically, leaf length, leaf width, and petiole length increased by 104%, 22%, and 28%, respectively, in OE-2 compared to Col-0 (*p* < 0.05; Figure 3d). These results indicate that *AhGRF3b* overexpression promotes leaf growth, increasing blade length, width, and petiole length.

To further investigate the function of *AhGRF3b* under cold stress, transgenic lines (OE-2 and OE-6) and Col-0 plants were treated at 0 °C for 7 days, and their growth status was assessed. Prior to cold treatment, no noticeable morphological differences were observed among the genotypes (Figure 3c and Figure S1). However, after 7 days at low temperature, Col-0 plants exhibited wilting and inhibited growth, whereas both *AhGRF3b*-OE-2 and *AhGRF3b*-OE-6 plants maintained relatively healthy growth (Figure 3c and Figure S1). Notably, after 7 days of 0 °C treatment, the fresh weight of OE-2 and OE-6 plants increased by 158% and 146%, respectively, compared to Col-0 (*p* < 0.05; Figure 3e). Effect size analysis further demonstrated that these differences were extremely strong (Cohen’s d = 11.6 for OE-2 vs. Col-0; d = 6.3 for OE-6 vs. Col-0).

In summary, overexpression of *AhGRF3b* not only significantly promotes leaf growth in *Arabidopsis* but also enhances its tolerance to low-temperature stress.

### 2.4. Interactions Between AhGRF3b and the Proteins AhCAT1, AhACR11, AhCAX3, and AhPAO4

**Figure 4:**
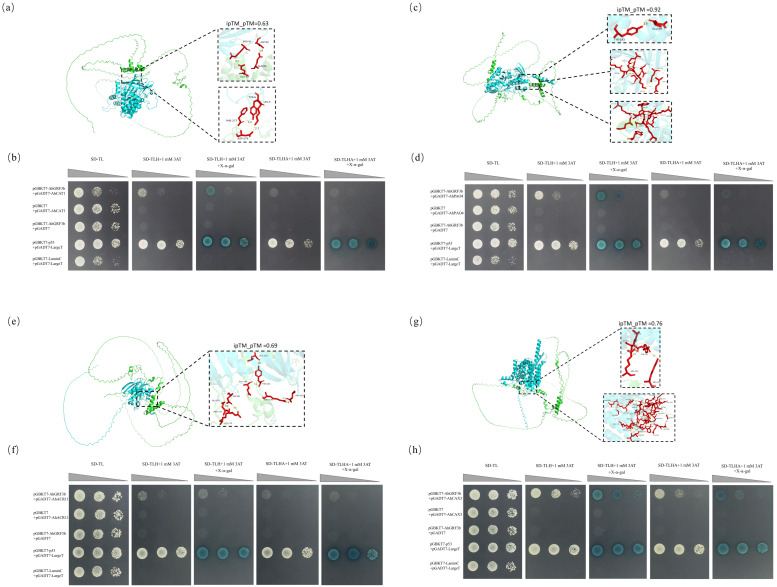
Interaction analysis of AhGRF3b with various proteins using yeast two-hybrid assays. (**a**,**c**,**e**,**g**) show the predicted 3D structures of AhGRF3b with its interacting proteins, highlighting the interface regions (Black dashed box). Green represents the AhGRF3b protein structure, blue indicates the interacting partner proteins (e.g., AhCAT1, AhPAO4, AhACR11, AhCAX3), and red highlights the binding interfaces or interacting residues (hydrogen bonds and salt bridges) between AhGRF3b and its partner proteins, and yellow marks the specific interaction sites or contact surfaces between AhGRF3b and its partner proteins. (**b**,**d**,**f**,**h**) depict the results of yeast two-hybrid assays, where combinations of bait and prey plasmids were co-transformed into *Saccharomyces cerevisiae*. The growth of yeast colonies on selective media (SD-TL, SD-TLH + 1 mM 3-AT, SD-TLH + 1 mM 3-AT, SD-TLH + 1 mM 3-AT) indicates interactions between AhGRF3b and its partners. The positive interaction is indicated by growth on the selective medium (blue color), while negative controls (empty plasmids) show no growth (white). Plasmid combinations tested include pGBKT7-AhGRF3b with pGADT7-AhCAT1, pGADT7-AhCAX3, pGADT7-AhPAO4, and pGADT7-AhACR11. Each interaction assay was performed in three independent biological replicates.

**Figure 5:**
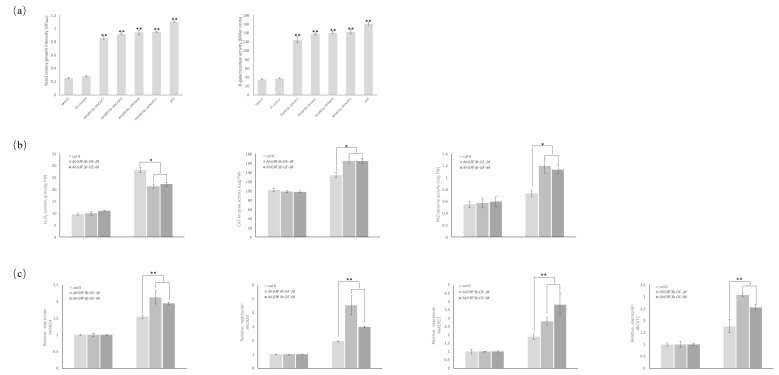
Quantitative assessment of AhGRF3b protein interactions and physiological responses in transgenic plants (**a**) Quantitative assessment of the yeast two-hybrid (Y2H) system interactions based on yeast colony optical density (OD_600_) and β-galactosidase activity. Data are presented as mean ± SD (*n* = 3), and analyzed by one-way ANOVA followed by Tukey’s HSD test. Asterisks indicate significant differences (** *p* < 0.01). (**b**) Measurement of physiological indices in Col-0 and transgenic *Arabidopsis* lines (*AhGRF3b-OE-2* and *AhGRF3b-OE-6*) under low-temperature stress. Shown are ROS contents, as well as the enzymatic activities of polyamine oxidase (PAO) and catalase (CAT). Data are presented as mean ± SD (*n* = 3), and analyzed by one-way ANOVA followed by Tukey’s HSD test. Asterisks indicate significant differences (* *p* < 0.05). (**c**) Quantitative RT-PCR analysis of *AhCAT1*, *AhPAO4*, *AhACR11*, and *AhCAX3* transcript levels in WT, *AhGRF3b-OE-2*, and *AhGRF3b-OE-6* plants. Expression levels were normalized to an internal reference gene. Data are presented as mean ± SD (*n* = 3), and analyzed by one-way ANOVA followed by Tukey’sHSD test. Asterisks indicate significant differences (** *p* < 0.01).

First, three-dimensional homology modeling analysis supported that AhGRF3b interacts with AhCAT1 (Catalase 1) through 5 hydrogen bonds and 3 salt bridges (Figure 4a; Table S1). In addition, AhGRF3b interacts with AhPAO4 (probable polyamine oxidase 4) via 19 hydrogen bonds and 15 salt bridges (Figure 4c; Table S1); with AhACR11 (ACT Domain Repeat protein) through 7 hydrogen bonds and 4 salt bridges (Figure 4e; Table S1); and with AhCAX3 (vacuolar cation/proton exchanger 3) at even more binding sites, forming a total of 30 hydrogen bonds and 14 salt bridges (Figure 4g; Table S1). To further validate these computational predictions, yeast two-hybrid assays were performed. In these experiments, the negative control grew only on SD-TL medium, whereas the positive control was able to grow on all selective media and displayed blue coloration on X-α-gal plates. Similarly, the experimental groups (pGBKT7-AhGRF3b + pGADT7-AhCAT1/AhPAO4/AhACR11/AhCAX3) (Figure 4b,d,f,h) also grew on all selective media and exhibited blue coloration on X-α-gal plates, confirming strong interactions between AhGRF3b and these proteins. Quantitative analysis based on yeast colony optical density (OD_600_) and β-galactosidase activity showed that all four interaction pairs exhibited significantly higher values than the negative controls (*p* < 0.01) (Figure 5a), confirming strong interactions between AhGRF3b and these proteins.

Furthermore, physiological and molecular assays provided functional support for these interactions. Under cold stress, ROS content in OE-2 and OE-6 plants decreased by ~24% and ~21%, respectively, compared to Col-0 (*p* < 0.05). Meanwhile, CAT activity increased by ~23% in both OE-2 and OE-6, and PAO activity increased by ~63% and ~55%, respectively (Figure 5b), suggesting that AhGRF3b may enhance ROS scavenging capacity by interacting with ROS-related proteins. Consistent with this, quantitative RT-PCR analysis demonstrated that the transcript levels of *AhCAT1*, *AhPAO4*, *AhACR11*, and *AhCAX3* were significantly upregulated in the *AhGRF3b*-overexpressing lines compared with WT (Figure 5c). These results indicate that AhGRF3b not only physically interacts with these proteins but may also regulate their expression, thereby coordinating ROS homeostasis, ion transport, and metabolic balance under cold stress.

In summary, the interactions of AhGRF3b with AhCAT1, AhPAO4, AhACR11, and AhCAX3 were verified through both three-dimensional modeling and yeast two-hybrid assays, and their functional relevance was supported by physiological and molecular analyses. Together, these findings suggest that AhGRF3b may play an important regulatory role in mediating protein–protein associations and enhancing cold stress adaptation in peanut.

## 3. Discussion

As plant-specific transcription factors, GRFs play crucial roles in crop growth, development, and adaptation to abiotic stresses. Recent studies have gradually demonstrated that *GRF* genes not only function in processes such as leaf development, floral organ formation, and seed development, but also participate in plant responses to abiotic stresses through interactions with multiple signaling pathways and regulatory networks [10]. In this study, we focused on the peanut gene *AhGRF3b* and combined transgenic functional verification, subcellular localization, physiological assays, and protein–interaction analyses to elucidate its mechanistic roles in leaf development and cold-stress response.

The evolution and functional diversification of *GRF* genes are also noteworthy. Previous comparative analyses in plants have shown that the *GRF* gene family has undergone significant diversification and expansion across species, closely associated with enhanced environmental adaptability [10,29]. Phylogenetic studies in multiple plant species further support both the conserved and diversified functions of GRFs, suggesting that the multi-member *GRF* family in peanut may have distinct roles in stress adaptation [10,30]. For example, *AhGRF3b* identified in this study may primarily function in leaf development and cold-stress response, while other family members could play more prominent roles under salt or drought conditions.

In terms of crop growth regulation and leaf development, GRFs play vital roles across plant species. Studies in rice have shown that *OsGRF4* markedly increases yield by promoting cell division and coordinating nitrogen metabolism, highlighting the potential of GRFs in crop improvement [31]. In peanut, our study demonstrated that overexpression of *AhGRF3b* significantly promoted leaf expansion, suggesting that GRFs are conserved regulators of vegetative organ growth in both monocot and dicot species. Quantitatively, in *A. thaliana*, *AhGRF3b*-OE-2 plants exhibited a 2.04-fold increase in blade length, a 1.22-fold increase in blade width, and a 1.28-fold increase in petiole length compared with Col-0 (*p* < 0.05; Figure 3b,d). This phenotype parallels observations in other species: overexpression of *PeGRF9* in eucalyptus significantly increased leaf area in *Arabidopsis* [32], while *PbGRF18* in pear reduced plant height but enlarged leaf area [33]. Mechanistically, GRFs—including AhGRF3b—have been reported to act with the GIF co-regulator and to influence cell-cycle genes such as CYCD3 in other species, suggesting a conserved GRF–GIF module that controls leaf size; although these direct targets were not tested here for peanut, our quantitative phenotypes are consistent with this model. Together, these findings provide robust quantitative evidence and support a conserved mechanistic framework in which GRFs promote leaf development by coordinating cell division and expansion.

With respect to cold stress, this study demonstrated that *Arabidopsis* plants overexpressing *AhGRF3b* exhibited better growth performance and higher fresh weight under 0 °C treatment. Importantly, physiological measurements demonstrated that transgenic lines accumulated lower levels of ROS but showed higher activities of CAT and PAO compared with wild type (Figure 5b). These results suggest that *AhGRF3b* enhances cold tolerance partly by boosting ROS scavenging systems. Previous evidence has shown that GRFs interact with DELLA proteins during cold responses, participating in the regulation of growth balance under low-temperature stress [34]. In tropical crops such as pitaya, *HpGRF8/9/10* genes were significantly upregulated under cold conditions, further indicating the general role of GRFs in cold adaptation [35]. Taken together with our findings, it can be inferred that *AhGRF3b* promotes plant survival under low-temperature environments by regulating both growth and oxidative stress pathways.

More importantly, this study demonstrated that AhGRF3b interacts with multiple proteins, including CAT, CAX, PAO, and ACR, which are functionally linked to ROS metabolism and ion homeostasis. The CAT family plays a central role in H_2_O_2_ scavenging and ROS homeostasis [36,37], and the enhanced CAT activity observed in AhGRF3b-overexpressing plants supports this interaction. Interestingly, in sweet cherry, overexpression of PavGRF5 increased ROS accumulation and reduced cold tolerance [38], highlighting that the GRF–ROS relationship can be bidirectional. Our results suggest that AhGRF3b positively contributes to ROS detoxification by both interacting with CAT proteins and upregulating their transcript levels (Figure 5b).

On the other hand, the *CAX* genes are key regulators of calcium signaling. Although overexpression of *CAX1* and *CAX3* can lead to growth retardation and necrosis [39], the upregulation of *AhCAX3* in *AhGRF3b*-overexpressing lines suggests that this interaction may help fine-tune ion homeostasis during cold stress. Similarly, *PAO* genes regulate polyamine metabolism and thereby ROS levels under stress conditions [40]; the elevated PAO activity in transgenic lines supports a role of *AhGRF3b* in activating this pathway. In addition, ACR proteins contribute to chlorophyll biosynthesis and photosynthetic efficiency [41]. The observed increase in *AhACR11* expression indicates that *AhGRF3b* may also improve photosynthetic energy supply, which is critical for sustaining growth under cold stress.

In summary, the functional characterization of *AhGRF3b* demonstrated in this study has multilayered significance. On one hand, this gene directly enhances photosynthetic capacity and growth potential by promoting leaf development; on the other hand, through its interactions with proteins such as CAT, CAX, PAO, and ACR, and by positively regulating their transcription, it indirectly modulates ROS metabolism, calcium signaling, and photosynthetic efficiency. Collectively, these mechanisms contribute to improved cold tolerance in transgenic plants. These findings not only deepen our understanding of the molecular basis of GRFs but also provide promising genetic resources for molecular breeding of cold-tolerant peanut varieties. Future research may leverage CRISPR/Cas9-mediated genome editing to precisely regulate *AhGRF3b* expression or modify its allelic variants, thereby enhancing stress adaptability without compromising growth. In addition, pyramiding *AhGRF3b* with other stress-responsive regulators such as *CBF/DREB*, *NAC*, or *WRKY* genes could yield peanut lines with enhanced cold tolerance and higher productivity. Nevertheless, potential risks–including growth–defense trade-offs, unintended impacts on other stress pathways, and biosafety or regulatory issues—should be carefully evaluated before field application.

## 4. Materials and Methods

### 4.1. Degradome Library Construction and Sequencing

To identify the potential targets, young leaves from peanut cultivar Fenhua 8 at the three-leaf developmental stage were collected and immediately frozen in liquid nitrogen for degradome library construction and deep sequencing. Through preprocessing, clean tags were generated. Then, clean tags were classified by alignment with GenBank, Rfam database, and miRNA database. Next, the reads were mapped to the *A. hypogaea* cv. Tifrunner reference genome (assembly Tifrunner gnm1 (v1), GenBank accession GCF_003086295.2, NCBI Annotation Release 101) (Table S2). The sense strand of peanut cDNA was used to predict miRNA cleavage sites using CleaveLand pipeline [42]. Based on the abundance of degradome tags at each cleavage site, transcripts were classified into five categories (0–4) according to established criteria. Category 0 represents the highest confidence, where the cleavage site has the most abundant tag and a single maximum. Category 1 has multiple sites with maximum abundance. Category 2 and 3 correspond to sites with tag abundance above or below the transcript median, respectively. Category 4 indicates sites supported by only one read. These categories reflect decreasing confidence in miRNA-guided cleavage (Table S3). Consistent with previous studies, ahy-miR396 indeed targets the *AhGRF1* and *AhGRF3b* genes (Table S3).

### 4.2. Western Blot

Leaf tissues were snap-frozen in liquid nitrogen and ground into fine powder. The samples were mixed with 2 × SDS loading buffer, boiled at 100 °C for 10 min, and centrifuged at 10,000× *g* for 10 min. The supernatant was separated on 12% polyacrylamide gels (1 mm thick) using the Laemmli buffer system (Tris–glycine–SDS). Electrophoresis was performed at 100 V for 1.5 h, and the proteins were transferred onto PVDF membranes (Bio-Rad, Hercules, CA, USA) using a wet transfer system (25 V, 90 min). A Precision Plus Protein™ Dual Color Standards ladder (Bio-Rad, USA) was used as the molecular weight reference. Membranes were blocked with 5% non-fat milk for 30 min at room temperature, followed by incubation with the primary anti-FLAG antibody (Abmart, Shanghai, China) for 2 h and the secondary antibody (Abmart, Shanghai, China) for another 2 h. Protein bands were visualized using chemiluminescence (Tanon, Shanghai, China), and band intensities were quantified with ImageJ software using β-Actin (Abmart, Shanghai, China) as the housekeeping loading control. For transient expression verification, three independent biological replicates were performed. AhGRF3b was cloned into the pCAMBIA1300 vector under the CaMV 35S promoter and fused with a C-terminal 3×FLAG tag to facilitate immunodetection. The C-terminal FLAG tag orientation was chosen to avoid interference with the N-terminal DNA-binding domain of AhGRF3b. Total proteins were extracted, and FLAG-tagged AhGRF3b was detected using the anti-FLAG antibody.

### 4.3. Cloning and Genetic Transformation of the AhGRF3b Gene

Based on the full-length coding sequence of *AhGRF3b*, specific amplification primers were designed. Total RNA was extracted from *A. hypogaea* (cv. Fenhua 8) young leaves using TRIzol™ Reagent (Invitrogen, Carlsbad, CA, USA) following the manufacturer’s protocol. RNA purity and concentration were measured with a NanoDrop spectrophotometer, showing A260/A280 = 1.98 ± 0.03, and integrity was confirmed using an Agilent 2100 Bioanalyzer with RIN ≥ 7.5. First-strand cDNA was synthesized from 1 µg total RNA using the PrimeScript™ RT reagent Kit with gDNA Eraser (Takara, Dalian, China) with a mixed priming method (oligo(dT)_18_ and random hexamers). The reverse transcription was carried out at 37 °C for 15 min, followed by enzyme inactivation at 85 °C for 5 s, and a no-RT control was included to verify the absence of genomic DNA contamination. Using the cDNA as a template, polymerase chain reaction (PCR) amplification was performed, and the full-length coding sequence of the *AhGRF3b* gene was successfully cloned. The cloned gene was then inserted into the pCAMBIA1300 vector via Gateway recombination technology, and the recombinant vector was introduced into *A. tumefaciens* strain GV3101.

Gateway cloning and construct validation. The *AhGRF3b* ORF was PCR-amplified with attB adapters and recombined into pDONR221 via BP Clonase™ II to generate the entry clone (attL1/attL2), followed by LR recombination into the destination vector pCAMBIA1300-35S::AhGRF3b-3×FLAG (C-terminal) using LR Clonase™ II. The attB adapter sequences appended to the primers were: attB1 (5′-GGGGACAAGTTTGTACAAAAAAGCAGGCT-3′) and attB2 (5′-GGGGACCACTTTGTACAAGAAAGCTGGGT-3′). Recombination proceeded through attB × attP → attL (BP) and attL × attR → attB (LR) reactions. Frame and orientation relative to the C-terminal 3×FLAG tag were verified by Sanger sequencing across the promoter–attB1–5′ UTR–start codon junction and the coding sequence–attB2–FLAG–stop junction. In addition, colony PCR with vector- and insert-specific primers confirmed insert size, and restriction analysis with diagnostic enzymes yielded the expected fragment sizes. Restriction-free validation was ensured by sequencing of the entire ORF and both att junctions. Three independent, sequence-verified LR clones were used for downstream transient expression and transgenic assays.

Transformation of *Arabidopsis* (ecotype Col-0) was performed using the *Agrobacterium*-mediated floral-dip method [43,44]. The recombinant strain *A. tumefaciens* GV3101 harboring the construct pCAMBIA1300-35S::*AhGRF3b*-3×FLAG was cultured in LB medium containing rifampicin (50 µg/mL) and hygromycin B (25 µg/mL) until OD_600_ ≈ 0.8. Bacterial cells were collected by centrifugation and resuspended in infiltration buffer (5% sucrose, 0.05% Silwet L-77). Flowering *Arabidopsis* plants were dipped into the bacterial suspension for 30 s with gentle agitation, then covered with plastic wrap for 24 h to maintain humidity. Transformed seeds (T_1_ generation) were selected on half-strength Murashige and Skoog (½ MS) medium supplemented with hygromycin B (25 µg/mL) as the selection marker for pCAMBIA1300. Resistant seedlings were confirmed by PCR using gene-specific primers. Hygromycin-resistant seedlings were transferred to soil for further propagation, and homozygous T_3_ lines were used for subsequent experiments.

### 4.4. Subcellular Localization

Three-week-old tobacco plants with well-developed leaves were selected, and *A. tumefaciens* GV3101 strains harboring the *AhGRF3b* recombinant vector were infiltrated into the leaves using infiltration buffer (10 mM MgCl_2_, 10 mM MES, pH 5.6, and 100 µM acetosyringone) at OD_600_ ≈ 0.8. After infiltration, plants were incubated at 25 °C, 60–70% relative humidity, under 16 h light/8 h dark photoperiod for 48 h to allow for protein expression and fluorescent signal recovery. Leaf samples were then observed under a confocal laser scanning microscope (Leica TCS SP8, Leica Microsystems, Wetzlar, Germany) to determine subcellular localization. All experiments were performed in three independent biological replicates.

### 4.5. Physiological Parameters and Cold Tolerance Assessment of Transgenic Plants

Using Columbia wild-type (Col-0) plants as the control and *AhGRF3b*-overexpressing *Arabidopsis* plants as the experimental group, at least 15 plants were included for each genotype. well-established in vitro seedlings were transplanted into soil and cultivated under conditions of 24 °C with a 16 h light/8 h dark photoperiod. Under normal growth conditions, the phenotypic characteristics of the transplanted seedlings were observed and recorded, while physiological parameters such as leaf length, leaf width, and petiole length were measured, followed by statistical analysis of the data. Subsequently, 20-day-old plants were transferred to low-temperature conditions (0 °C) for 7 days with the same photoperiod. After treatment, plant phenotypes were again recorded, and fresh weight was measured to compare the performance of different genotypes under cold stress.

### 4.6. Yeast Two-Hybrid (Y2H) Assay

Protein 3D models were constructed using SWISS-MODEL based on templates with maximal sequence identity and coverage (≥30%, ≥50%, and ≥80% thresholds corresponding to expected accuracies of ~80%, ~95%, and representative structures, respectively). Template PDB IDs, sequence identities, and coverages are summarized in Table S4. Protein–protein docking was performed using MEGADOCK v4.1.4, and seven evaluation metrics (Total, ELEC, RecACE, rPSC, rPSC_gain, rPSC_penalty, LCARMSD) were used to assess interaction quality. Complexes were further refined with HDOCK, and confidence scores ≥ 0.7 were considered indicative of high-confidence binding. For AlphaPulldown-generated complexes, iptm_ptm confidence values (>0.5) were reported. Hydrogen-bonding interactions were analyzed using PyMOL and were displayed in Table S1. The three-dimensional structure of the AhGRF3b protein was predicted using SWISS-MODEL. Functional annotation of the peanut whole-genome genes was performed using the eggNOG v5.0 database (http://eggnog5.embl.de/#/app/home, accessed on 10 March 2024). After preliminary screening, fine screening, and validation, four candidate genes—*AhCAT1, AhCAX3, AhPAO4*, and *AhACR11*—were selected for subsequent analysis.

To investigate potential protein–protein interactions, docking models between AhGRF3b and the candidate proteins were visualized. The three-dimensional structures of the proteins were modeled using AlphaFold 2 (version 2.3.1, DeepMind Technologies, London, UK) [45], followed by protein–protein docking using HDOCK (version 1.1, Huazhong University of Science and Technology, Wuhan, China) [46]. Multiple protein–protein complex structures were obtained and ranked based on confidence scores (≥0.7). The top-ranked conformations were analyzed for binding site information using the PISA server (https://www.ebi.ac.uk/pdbe/pisa/, accessed on 10 March 2024), and the identified binding sites were visualized using PyMOL (version 2.5, Schrödinger, LLC, New York, NY, USA).

The coding sequence of *AhGRF3b* was cloned into the pGBKT7 vector, while AhCAT1, AhCAX3, AhPAO4, and AhACR11 were cloned into the pGADT7 vector. The constructed plasmids were co-transformed into *S. cerevisiae* strain AH109. For controls, pGBKT7-p53 + pGADT7-largeT (a known interacting protein pair provided by the kit) were used as positive control, while pGBKT7-laminC + pGADT7-largeT and empty vector combinations (BD or AD fused to GAL4 domains only) served as negative controls.

Selective media composition: SD minimal media contained yeast nitrogen base without amino acids (6.7 g/L), glucose (20 g/L) as the carbon source, and agar (20 g/L) for solid media. The dropout components were adjusted as follows: SD/-Trp-Leu (SD-TL), SD/-Trp-Leu-His (SD-TLH), and SD/-Trp-Leu-His-Ade (SD-TLHA). When indicated, 1 mM 3-AT and/or 40 μg/mL X-α-gal were added. All media were antibiotic-free due to auxotrophic selection. For each interaction combination, yeast cells were plated on selective media (SD-TL, SD-TLH, and SD-TLHA) and incubated at 30 °C for 3–5 days.

Quantitative assessment of Y2H interactions was performed to complement visual inspection. Yeast colonies were resuspended in sterile water, and growth intensity was measured as optical density at 600 nm (OD_600_). β-galactosidase activity was further determined using the ONPG assay (Clontech Yeast Protocols Handbook). Each assay was conducted with at least three independent biological replicates. Data were expressed as mean ± SD and analyzed using one-way ANOVA followed by Tukey’s HSD test.

## 5. Conclusions

In summary, this study identified *AhGRF3b* as a key regulator of peanut leaf development and cold stress response. Functional and molecular analyses demonstrated that *AhGRF3b* promotes leaf expansion by enhancing cell proliferation and is directly repressed by ahy-miR396. Transgenic experiments further supported that *AhGRF3b* overexpression increases leaf size and improves tolerance to low-temperature stress. Protein interaction assays indicated that *AhGRF3b* cooperates with ROS scavenging- and ion homeostasis-related proteins, suggesting a broader regulatory role in stress adaptation. Collectively, these findings suggest that the ahy-miR396/*AhGRF3b* module plays an important regulatory role in peanut growth and abiotic stress responses, and may provide useful preliminary resources for future molecular breeding of cold-tolerant peanut cultivars.

## Data Availability

The data presented in this study are available in this article. The raw data of degradome sequencing were submitted to the NCBI database with the bioproject ID: PRJNA1312356.

## Supplementary Materials

The following supporting information can be downloaded at: https://www.mdpi.com/article/10.3390/plants14203203/s1, Table S1: Predicted salt bridges and hydrogen bonds from docking between AhGRF3b protein and interacting proteins; Table S2: Statistics of degradome reads in Degradome Library; Table S3: Statistical Overview of Degradome-Identified miRNA Targets; Table S4 Predicted interactions between AhGRF3b and candidate bait proteins; Table S5: Primer information; Figure S1: Phenotypic comparison before and after low-temperature treatment.

## Abbreviations

The following abbreviations are used in this manuscript:

GRFs	Growth-regulating factors
CAT	Catalase
PAO	Probable Polyamine Oxidase
ACR	ACT Domain Repeat protein
CAX	Vacuolar cation/proton exchanger
