# Supplementary material for: Targeted Regulation of AhGRF3b by ahy-miR396 Modulates Leaf Growth and Cold Tolerance in Peanut
Source: Plants (Basel). 2025 Oct 18;14(20):3203. doi: 10.3390/plants14203203 (PMC12567176; doi:10.3390/plants14203203)
Supplement: Supplementary file 1 [file plants-14-03203-s001.zip › Supplementary Materials.pdf]

Supplementary Materials

Targeted Regulation of *AhGRF3b* by ahy-miR396 Modulates Leaf Growth and Cold Tolerance in Peanut

Xin Zhang [1], Qimei Liu [2], Xinyu Liu [3], Haoyu Lin [3], Xiaoyu Zhang [2], Rui Zhang [4], Zhenbo Chen [4], Xiaoji Zhang [4], Yuexia Tian [1], Yunyun Xue [1], Huiqi Zhang [1], Na Li [1], Pingping Nie [3,*]; and Dongmei Bai [1,4,*]

*** Correspondence:**
PingPing Nie: niepingping@uzz.edu.cn
Dongmei Bai: sxndjzsbdm@sxau.edu.cn

1 Supplementary Figures and Tables

1.1 Supplementary Figures

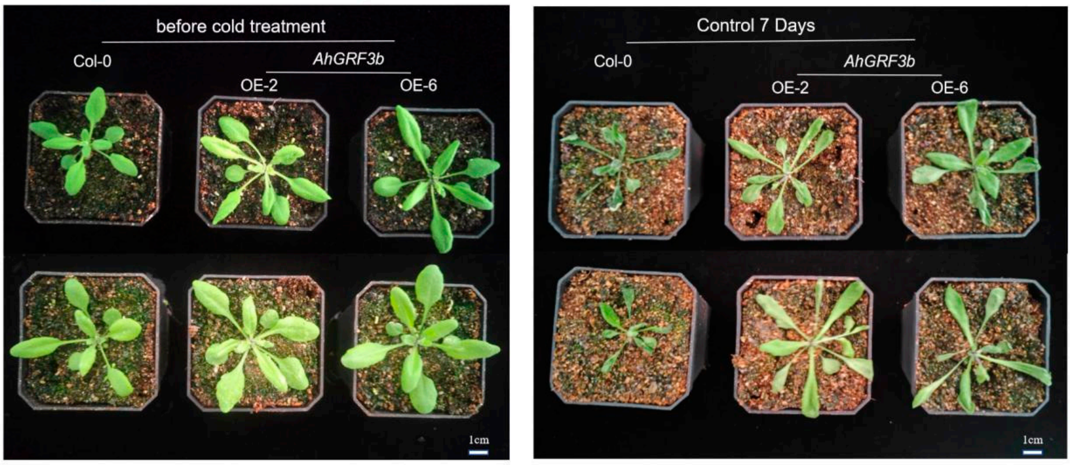

Figure S1. Phenotypic comparison before and after low-temperature treatment. Phenotypic differences of *AhGRF3b*-overexpressing plants under cold stress. The left panel shows plants before treatment, and the right panel shows plants after 7 days at 0 °C. Compared with wild-type Col-0, the *AhGRF3b*-overexpressing lines (OE-2, OE-6) maintained better growth and enhanced cold tolerance following cold treatment. Scale bar = 1 cm.

1.2 Supplementary Tables

Table S1: Predicted salt bridges and hydrogen bonds from docking between AhGRF3b protein and interacting proteins;

Table S2: Statistics of degradome reads in Degradome Library;

Table S3: Statistical Overview of Degradome-Identified miRNA Targets:

Table S4: Predicted interactions between AhGRF3b and candidate bait proteins

Table S5: Primer information;
