# Supplementary figures and images for: Targeted Regulation of AhGRF3b by ahy-miR396 Modulates Leaf Growth and Cold Tolerance in Peanut
Source: Plants (Basel). 2025 Oct 18;14(20):3203. doi: 10.3390/plants14203203 (PMC12567176; doi:10.3390/plants14203203)

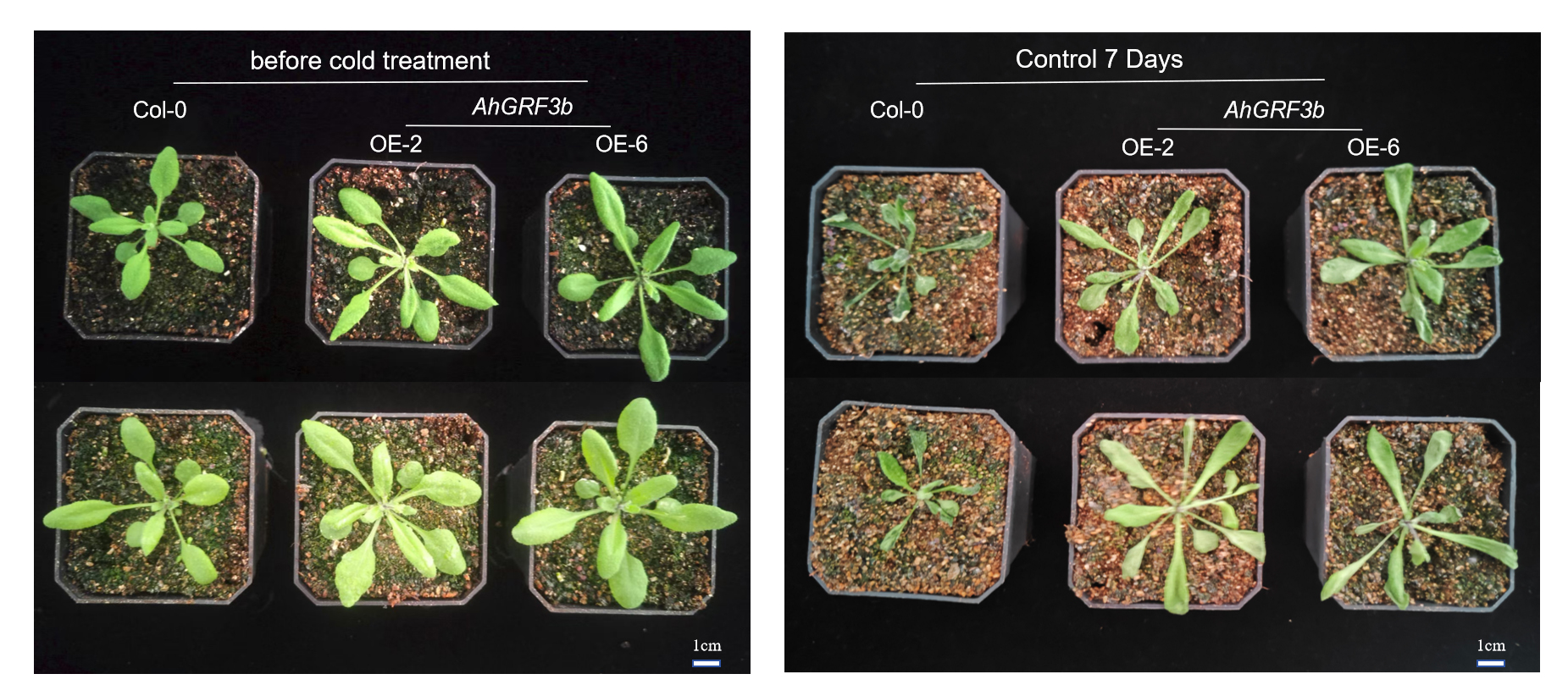

Supplement: Supplementary file 1 [file plants-14-03203-s001.zip › Figure S1.jpg]
